# From Public Health Policy to Impact for COVID-19: A Multi-Country Case Study in Switzerland, Spain, Iran and Pakistan

**DOI:** 10.3389/ijph.2022.1604969

**Published:** 2022-08-31

**Authors:** Maryam Tavakkoli, Aliya Karim, Fabienne Beatrice Fischer, Laura Monzon Llamas, Azam Raoofi, Shamsa Zafar, Carmen Sant Fruchtman, Don de Savigny, Amirhossein Takian, Marina Antillon, Daniel Cobos Muñoz

**Affiliations:** ^1^ Department of Public Health and Epidemiology, Swiss Tropical and Public Health Institute (Swiss TPH), Basel, Switzerland; ^2^ University of Basel, Basel, Switzerland; ^3^ Independant Consultant, Gran Canaria, Spain; ^4^ Department of Health Management, Policy & Economics, School of Public Health, Tehran University of Medical Sciences, Tehran, Iran; ^5^ Health Equity Research Centre, Tehran University of Medical Sciences, Tehran, Iran; ^6^ Fazaia Medical College, Islamabad, Pakistan; ^7^ Department of Global Health & Public Policy, School of Public Health, Tehran University of Medical Sciences, Tehran, Iran

**Keywords:** COVID-19, pandemic, governance, health system, public healh, COVID-19 restrictions, cross-country comparison, policy responses

## Abstract

**Objectives:** With the application of a systems thinking lens, we aimed to assess the national COVID-19 response across health systems components in Switzerland, Spain, Iran, and Pakistan.

**Methods:** We conducted four case studies on the policy response of national health systems to the early phase of the COVID-19 pandemic. Selected countries include different health system typologies. We collected data prospectively for the period of January–July 2020 on 17 measures of the COVID-19 response recommended by the WHO that encompassed all health systems domains (governance, financing, health workforce, information, medicine and technology and service delivery). We further monitored contextual factors influencing their adoption or deployment.

**Results:** The policies enacted coincided with a decrease in the COVID-19 transmission. However, there was inadequate communication and a perception that the measures were adverse to the economy, weakening political support for their continuation and leading to a rapid resurgence in transmission.

**Conclusion:** Social pressure, religious beliefs, governance structure and level of administrative decentralization or global economic sanctions played a major role in how countries’ health systems could respond to the pandemic.

## Introduction

Since the World Health Organization (WHO) announced SARS-COV-2 as a public health emergency of international concern on 31 January 2020, countries have applied various strategies to control the spread of the virus [1, 2]. Health systems are key to the response to COVID-19, but are also highly vulnerable to collapse due to the demands posed by the rapid expansion of the demand for services [3–5], yet it was not until 18 April 2020 that the WHO’s Regional Office for Europe published its technical working guidance, Strengthening the Health System Response of COVID-19 [3], more than 4 weeks after the pandemic had been declared on March 11, 2020. However, many of its recommendations were similar to the principles in other documents for other outbreaks [6], and the recommendations were sufficiently generic to be adapted within different contexts.

However, both complexity theory and our experience tells us that there is no one-size-fits-all strategy. Inevitably, however, the WHO and country-level officials must glean the best approach from general principles of health and governance systems, past experience, and the demands of the population at the time, managing not only the expectations and principles mandated by their constituents and their constitutions, but also an unusual amount of uncertainty during an outbreak of an emerging pathogen [7–9].

The variability of responses to the pandemic and the interplay of different elements warrants approaches that account for complexities inherent to a country’s political, economic, and social context; their existing health systems structures; and disease dynamics. Understanding and managing this complexity through a systems lens is essential to enable governments to better adapt and respond to threats like the current pandemic [10].

Since the onset of the pandemic, case studies have been carried out to monitor country-specific response to the pandemic [11, 12]. In a study reviewing the preparedness in 177 countries, environmental seasonality, altitude and GDP per capita were identified as the main contextual factors influencing the COVID-19 infections rate [13]. However much uncertainty still exists about the extent to which these factors influence the desired outcome in countries [14–16].

We aimed to assess the influence of the political and health systems response on COVID-19 incidence in Switzerland, Spain (high-income countries in Europe region) and Iran, Pakistan (Middle-income countries in Eastern Mediterranean Region). The selected countries included different health system typologies (see countries’ health system profile in Table 1), whose governance around health care ranges from extensively decentralized systems in Switzerland and Spain to more centralized systems in Iran and Pakistan [17].

**TABLE 1:** Health systems profile (Switzerland, Spain, Iran and Pakistan, 2019) [17, 67].

Country	Population	Income level	Healthy life expectancy at birth (years)	UHC: Service coverage index^a^	Density of medical doctors (per 10k population)	Density of nursing and midwifery personnel (per 10k population)	Current health expenditure (%of GDP)	Compulsory health insurance (CHI) as % of current health expenditure (CHE)
Switzerland	8591	High	72.5	83	43.3	178.9	11.29	44
Spain	46,737	High	72.1	83	40.3	60.8	9.13	4
Iran	82,914	Upper-middle	66.3	72	15.8	20.8	6.71	35
Pakistan	216,565	Lower-middle	56.9	45	11.2	4.8	3.38	1

a Coverage of essential health services (defined as the average coverage of essential services based on tracer interventions that include reproductive, maternal, newborn and child health, infectious diseases, non-communicable diseases and service capacity and access, among the general and the most disadvantaged population). The indicator is an index reported on a unit-less scale of 0–100, which is computed as the geometric mean of 14 tracer indicators of health service coverage.

In this article, we provide an assessment of the implementation of the measures recommended by the WHO within the six building blocks of health systems. We qualitatively investigated the effect of the system responses over time and the influence of context-specific factors on the measures put in place by governments to contain the pandemic. Our study sheds light on the dynamic interaction of the health, social, economic and cultural systems and how they influenced the ability to manage the pandemic.

## Methods

We conducted four case studies to explore the national response to the COVID-19 pandemic from January through July 2020, corresponding to the “first wave.” To capture what measures were taken to prepare the system for the COVID-19 response in Switzerland, Spain, Iran, and Pakistan, we collected data on several interventions mentioned in the WHO’s technical guidance document to support countries strengthening the health system response to COVID-19 [3] within each health system component. We tracked the presence of these measures weekly on the basis of 17 indicators. We also collected qualitative data on the rationale and the political support for these measures.

We developed the first version of the data collection tool in Microsoft Excel. We pilot-tested this version by collecting information for 1 week. After discussion with key informants, we selected 17 indicators (Table 2) and tracked their implementation prospectively; these indicators encompass the thematic domains of governance, financing, health workforce, information, medicine and technology and service delivery.

**TABLE 2:** List of selected Indicators and domains of public health policy response to Covid-19 (Switzerland, Spain, Iran and Pakistan, January-July 2020).

**Governance**
Coordination mechanism created
Level of decentralization in COVID response in the health sector
**Finance**
Introducing emergency legislation to finance response to COVD-19. source, e.g. mobilized emergency reserve funds; reallocated from other budget lines; etc.
**Human resources**
Mobilizing and repurposing health workforce (e.g. reserves, retired staff, staff from other specializations, trained students, etc.)
**Information systems**
Media briefing at regular intervals
**Medical technologies and pharmaceuticals**
Ensuring emergency mechanisms are in place for procurement and registration of medicines and health technologies
**Service delivery**
Contact tracing
Screening on entry
**Preventive measures**
Quarantine/home isolation of COVID-19 patients
Quarantine/home isolation of suspected cases and contacts of confirmed patients
Announcement of preventive activities (personal hygiene)
Physical distancing
Restrictions on congregation
Closure of schools and other teaching facilities
Closure of bars, restaurants, sports venues
Lockdown
Border closure/Travel restriction

Teams of health systems researchers from Switzerland, Spain, Iran, and Pakistan volunteered to participate as key informants. We collected information on country responses from publicly available sources including official government documents (legislation, press releases, policy briefings); reports from different agencies in countries; and major media channels. Information was extracted in German, Spanish, Persian and Urdu in Switzerland, Spain, Iran and Pakistan respectively, and translated to English in the data matrices. Some data in all countries was available in English. Finally, two independent researchers performed data reviews and quality control for each country. To gain a comprehensive view of how the measures tracked with the case burden in each country, we developed a set of visuals side-by-side with two simple indicators of the epidemiological situation: the incidence and the basic or effective reproductive number (*R*_*e*_) by day, sourced from the COVID-19 Datahub using the associated R Package as an interface [18, 19]. We also graphed the weekly number of tests and the percent positivity; we opted for the weekly statistics rather than the daily statistics because daily statistics can be noisy (i.e. fewer tests take place on weekends, more cases are reported on Mondays). To understand whether there was significant relationship between the tests per million population and positivity rate we ran a Pearson Chi2 test.

The information collected was structured and analyzed around the domains of the health system. To gain a detailed, holistic view of the development of the response in each country, each of our key informants gave a narrative overview of the health commodities, restrictions, and economic response to the pandemic framed around the centralized or decentralized nature of governance.

## Results

**FIGURE 1:**
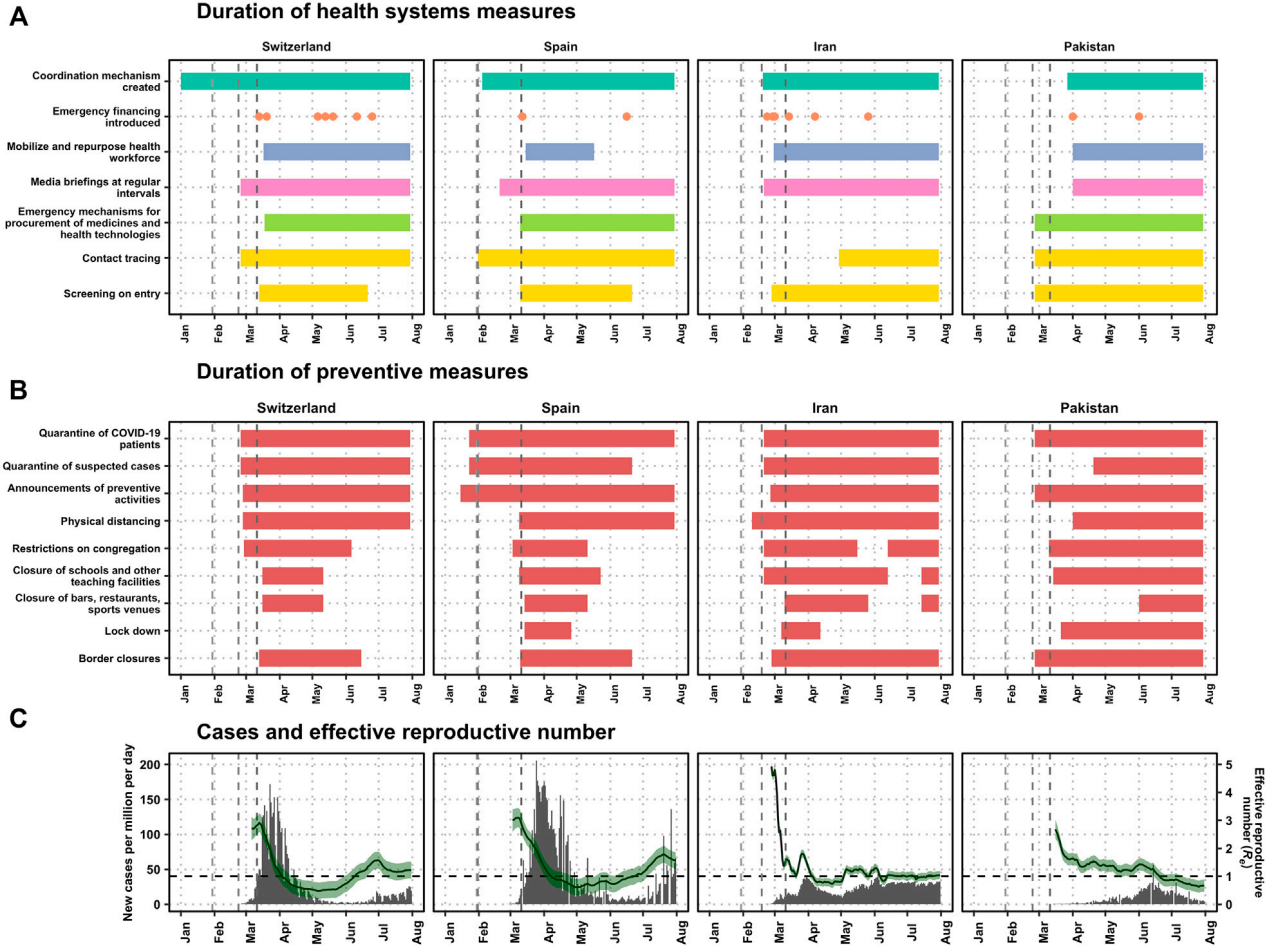
Timeline for public health policy response over cases and number of cases per country (Switzerland, Spain, Iran and Pakistan, January–July 2020). **(A)** The duration of the measures, organized by the six building blocks of health systems (Switzerland, Spain, Iran and Pakistan, January-July 2020). The first gray dashed lines represent the day that the World Health Organization declared the pathogen a subject of international concern and the second line represents the first day of a COVID-19 case was detected in each country: 23 February in Switzerland, 31 January in Spain, 18 February in Iran, and 24 February in Pakistan. In Spain, the day that the WHO declared the pathogen of international concern was 1 day before the first case was found in Spain, and therefore the difference between the first two lines is indistinguishable in the graph. The third gray line shows the day when the WHO declared the pandemic: 11 March 2020. **(B)** The duration of preventive policies (Switzerland, Spain, Iran and Pakistan, January-July 2020). **(C)** The number of cases and effective reproductive number (Switzerland, Spain, Iran and Pakistan, January-July 2020). The number of cases (in gray bars) and the estimated effective reproductive number (R, in green) with 95% confidence intervals as estimated by [18], and assuming a serial interval of 7 days. The dashed horizontal line in black shows R_e_ = 1, the threshold above which the pandemic is growing.

The data collection strategy yielded more than 100 data points over 8 months on the implementation of the different strategies. The detail of the sequence of the interventions in countries combined with the measures of disease progression can be seen in Figure 1 for Switzerland, Spain, Iran, and Pakistan (see Supplementary Appendix SA1 for list of information sources).

Visual inspection of the policy response in Figure 1 is complemented by the information about the policy response in Table 2 for the four countries. Our findings show that enactment of the public health policy responses coincided with the decrease in the transmission, expressed in terms of the effective reproductive number.

Multi-sectoral coordination committees began either before or immediately after the first case in all countries but Pakistan (Figure 1; Table 3). Initially the response was centralized at the national level in all four countries and then with increasing geographic heterogeneity in prevalence of COVID-19 cases within a country, more localized approaches were adopted.

**TABLE 3:** Describing selected Indicators and domains of public health policy response (Switzerland, Spain, Iran and Pakistan, January–July 2020).

Domain of response	Switzerland	Spain	Iran	Pakistan
Governance				
Coordination mechanism created	Legislation in place before the pandemic. The epidemics act “EpiA” clarifies the work-sharing and other coordination aspects between confederation and cantons during a crisis	The Inter-territorial Council of the National Health System, which is the government body of the health system, laid ground for the collaboration between the national and regional health authorities	In February 19,, the National Covid-19 Committee (NCC) led by the minister of health and medical education was established to achieve maximum coordination and inter-sectoral cooperation/Establishing a joint committee (the scientific sub-committee of the NCC) consisting of some deputies of the Ministry of Health and members of the parliamentary health commission	A multi-sectoral response was designed through the creation of the National Coordinating Council (NCC) to manage the epidemic in March 13. The NCC was headed by the Prime Minister alongside representatives from all relevant ministries. Subsequently, on March 27 the National Command and Operations Center (NCOC) was established, this civil-military constellation proved to be critical in fast-tracking logistics, information gathering, real‐time reporting and “smart” lockdowns
Level of decentralization in COVID response in the health sector	Policy-making in Switzerland is usually decentralized. Health care is mostly organized in cantonal level. During an epidemic, the epidemics act “EpiA”, allows the transfer of decision-making from sub-national to national levels through escalating steps from “normal”, over “special” to “extraordinary situation”	The state of alarm was declared on March 14, this conferred to the central Government full responsibility for implementing measures for COVID-19 crisis. Regional administrations retain operational management of health services	General regulations have been passed by the national committee, while provincial committees are obliged to pass specific regulations based on provinces’ situation in line with national committee regulations. National committee also announced the need for continuous monitoring and control over the measures of the provinces	The response initially in February and March was decentralized, as the provinces were independent. But after NCOC was established on March 27 the response was mainly central
Finance				
Introducing emergency legislation to finance response to COVD-19. Briefly describe source, e.g. mobilized emergency reserve funds; reallocated from other budget lines; etc.	In 2020. mobilized estimated CHF 70 to 80 billion from high level of liquidity but also incurrence of debt	A Royal decree approved on March 12 2020 to implement measures that allow exceptional mobilization of structural and contingency funds; Release of extra funds to support the education sector for COVID-19 crisis	Mobilizing $1,127,770,000 from the National Development Fund; allocating $176,229,885 by the government to the country’s health system; $62,362,297 foreign financial facilities to fight Corona; etc.	The initial shortage of health commodities and medical equipment in April and May was addressed by the disbursement of more than six billion Pakistani rupees (PKR) (US$ 37M) to buy equipment, ventilators and to upgrade hospital facilities. Additionally, state banks provided low-interest loans to hospitals to improve their case management capacity
Human resources				
Mobilizing and repurposing health workforce (e.g. reserves, retired staff, staff from other specializations, trained students, etc.)	National level: Non-emergency procedures have been prohibited March 21 - April 27, 2020. Other mobilization was organized largely on a cantonal or even hospital level. Cantons can request private institutions to provide their resources for COVID-19 support	Regulation to adopt measures for human resources management during the covid19 crisis. Some Autonomous Communities implemented measures to mobilize the health workforce to cope with the crisis	Reserving 5%–10% nursing staff from other wards of the hospitals for COVID-19 wards; Recruiting individuals who have capability for nursing, (i.e retirees, unemployed nurses, volunteers and interns); Invite nursing professionals, faculty members and post-graduate nursing students to counsel people via the 4030 hotline	A shortage of trained professionals in critical care units was observed in the beginning of the pandemic. Training programs were launched for health care staff
Information systems				
Media briefing intervals	Media releases/press conferences are done at irregular intervals, but several times a week. Special press conferences with specific topics (e.g. sport) are released additionally	From February, the Government released the latest update on the pandemic evolution and the implementation of different measures and policies, at daily press conferences	From February, ministry of health published daily reports of covid-19 statistics including new/total cases, deaths and laboratory tests	Daily media briefings by NCOC started in April and continued for a long time
**Medical technologies and pharmaceuticals**				
Ensuring emergency mechanisms are in place for procurement and registration of medicines and health technologies	In General, the supply of essential medical products is organized in COVID-19 Ordinances 2/3. Some selected smaller scale measures: i) Procurement can be done on a federal level via the military; ii) exceptions are made concerning legal requirements of medical products; iii) essential medicines are given out only in limited amounts; iv) mandatory reporting of ICU availabilities, PPE stocks etc.	There were mechanisms in place but they did not ensure the access to specific health technologies and PPEs	Due to economic pressures from sanctions and the ban on foreign exchange transactions, the possibility of importing medicines and health equipment was minimized. Therefore, the country developed and implemented mechanisms to encourage Iranian companies and factories to increase domestic production lines and achieve self-sufficiency	The NCOC provided vital PPE, oxygen supply systems and established COVID-19 care and treatment centers through National Disaster Management Authority
Service delivery				
**Identification of cases**				
Contact tracing	Contact tracing done by the cantons. Contact tracing app “SwissCovid app” piloted in June 2020	The contact tracing protocol classified “close contacts” or as possible, probable or confirmed cases; On May 9, the MoH published new guidelines for early detection of cases and contact tracing. Tracing workers would track down people who were closer than 2 m and for more than 15 min to suspected or confirmed cases	A mobile app (mask app) was developed for this purpose. But it was not widely used	Contact tracing conducted by rapid response team, including primary healthcare doctors, nurses and paramedics
			All people in contact with cases should be screened within 14 days after contact	
Screening on entry	Since March 13, travel from “risk countries” (neighboring Italy at the time) was restricted. This list was slowly expanded. On May 11, first travel restrictions were relaxed. Since June 2020, passengers from “risk countries” could have their temperature measured. From July 2020, travelers from “risk countries” need to quarantine for 10 days	Initially, after the detection of the first imported case on January 31, public health interventions were activated to detect cases coming from China; In March, travel bans were imposed from Italy and cruises from any origin; In May land borders closure measures were implemented	Inbound travelers from abroad were required to fill out an entry form/Prohibition of passenger entry into the aircraft without a mask/Screening before exist/airport public places disinfection, including terminals and aircraft/Develop a special procedure for protecting flight controllers/flight restriction	Initially screening only applied to travelers from China. Then extended to the pilgrims from Iran who were quarantined at Taftan border
**Preventive measures**				
Quarantine/home isolation of COVID-19 patients	Isolation of positive cases for 10 days	Cases with symptoms were isolated at home and followed up by a PHC team, or hospitalized if needed	Compulsory quarantine of infected people was approved by the NCC, its implementation was not monitored	Quarantine facilities were established in major cities in the early phases
Quarantine/home isolation of suspected cases and contacts of confirmed patients	Quarantine of close contacts of positive cases for 10 days	Initially, suspicious cases were isolated on arrival, and potential contacts investigated. During the state of alarm, symptomatic cases were isolated at home and potential contacts further investigated.	All people in contact with cases should be screened within 14 days after contact	Quarantine facilities were established in major cities in the early phases
Announcements of preventive activities (personal hygiene)	Public information campaign updated with new rules and recommendations (e.g., hand washing) at different intervals	Personal hygiene, physical distance and indoor preventive and hygienic measures	Personal hygiene protocols were recommended	After NCOC took the control national strategy for communication was developed
			Since July 5 wearing face mask became mandatory in public places	
Physical distancing	Initially 2 m, scaled back to 1.5 m	When the first community outbreak was declared, progressive physical distancing measures were implemented. After the state of alarm declaration, citizens were required to stay at home and use public roads just when carrying out specific activities	It was recommended but not mandatory	All preventive measures were communicated but not strictly followed
			Eid al-Fitr prayers was held outdoors of mosques	
			Introducing staggered office hours	
Restrictions on congregation	Congregations banned at various levels of stringency, e.g. prohibiting gatherings of more than five people	On March 10, sports events were limited to closed doors and, in regions with community transmission, events with more than 1000 people were banned. When the state of alarm started, citizens were required to stay at home and congregation was not allowed	Issuance of regulations by the government regarding restrictions on gatherings in high risk areas	Non-essential services such as educational institutions, government offices, markets, business centers, parks, etc., were closed
Closure of schools and other teaching facilities	Schools on all levels closed for 2 months. Step-wise reopening (Secondary level II, tertiary level and further education last), shift of decisions to cantons	Schools and universities were closed, first in the regions with community transmission, followed by application country-wide on March 12th. When the de-escalation plan started to be implemented, during the state of alarm, educational centers could open under particular circumstances	In the metropolis of Tehran and other red-zone cities: Closure of all universities, seminaries, educational centers, and libraries (the NCC scientific committee has classified the country into five zones according to the COVID-19 situation in each city: red, orange, yellow, blue, and white. In this classification, white zone is where no new COVID-19 cases are found, and the red zones are the cities with the most infected cities)	In March all the educational institutions, were closed to reduce the spread of COVID‐19
Closure of bars, restaurants, sports venues	Fully closed for 2 months, afterwards opening with restrictions (e.g. four people per table)	During the first months of state of alarm, hotels and restaurants had to close, except if they had been recruited to serve healthcare workers or truck drivers. In May, during the de-escalation plan, bars and restaurants in some regions could open with some restrictions. Professional sports competitions were allowed behind closed doors	Fully closed in red-zone cities. Re-opening with restrictions in lower risk zones	Fully closed during lockdown, afterwards opening with restrictions in lower risk areas
Lockdown	Not considered	Total lockdown started on March 14 and was progressively scaled back (with the de-escalation plan) until June 21	Lockdown was in place including closing businesses and government offices and inter-city and inter-province travel bans. Later, using a color coded scale, cities were classified into blue, yellow, orange, and red zones based on the COVID-19 infection rate. In red cities, only essential services were allowed to open. Inter-city travel was banned	After the low compliance with the initial decision on national lockdown for 2–3 months, prime minister ordered to reopen the economy and move to a strategy of contact tracing and “smart lockdown” in areas with high positivity ration
			Blue was the lowest threat with minimum restrictions	
Border closure/Travel restriction	Closure of borders/travel restrictions and stepwise reopening (first neighboring countries, Schengen area, then other countries)	Closure of borders/travel restrictions and stepwise reopening (first neighboring countries, Schengen area, then other countries)	Partial closure of borders/travel restrictions and stepwise reopening	Initially only china but later included other countries. Since March 2020, Pakistan suspended domestic and international flight operations and reopened the borders in stepwise manner

Emergency financing measures were introduced in all countries within 1 month of the first case (Figure 1; Table 3). The healthcare workforce was repurposed in all countries within 3 weeks except in Pakistan, where it took more than 1 month. Media briefings at regular intervals began within 2 months of the WHO declaration of COVID-19 as a disease of international concern; it began in February 2020 and after the report of the first case in Switzerland, Spain and Iran, but more than 1 month after the first case in Pakistan.

Emergency mechanisms for the procurement of medicines and health technologies began on the day that the first case was reported in Pakistan, after 3 weeks in Switzerland, and 1 month after the first case in Spain; these mechanisms were never put in place in Iran (Figure 1). Contact tracing began in Spain, Switzerland, and Pakistan on the day that the first case was reported in the country, and in Iran more than 2 months after the first case was reported in the country. Screening on entry to the country—symptom screening and tracing services—was mandated on the day of the first detection in Pakistan, on the week of the first detection in Iran, and within 3 weeks in Spain and Switzerland. Preventive measures were in place at different time intervals and intensity in each country.

**FIGURE 2:**
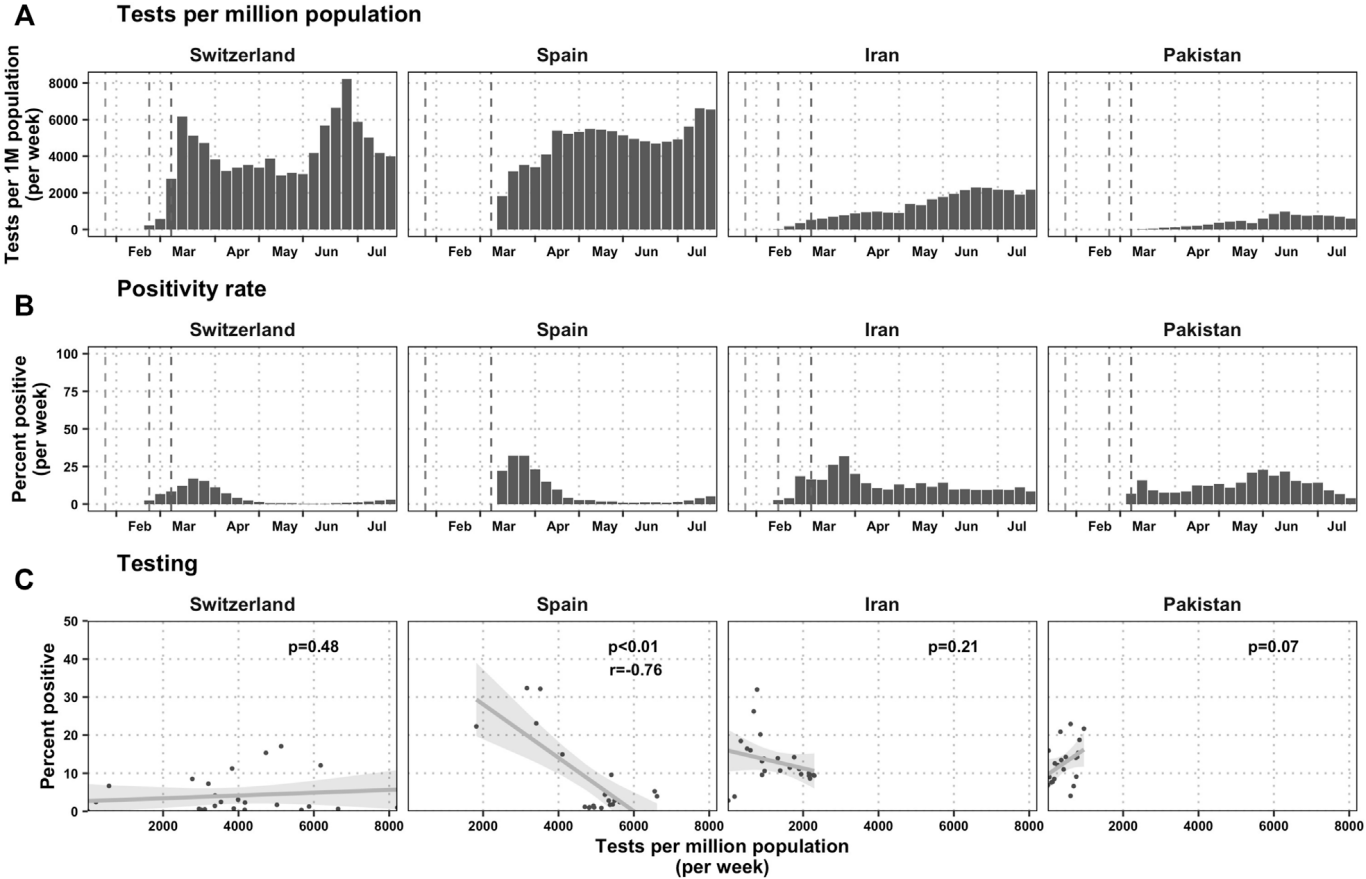
Testing statistics per country (Switzerland, Spain, Iran and Pakistan, January-July 2020). **(A)** Tests per million population. The first gray dashed lines represent the day that the World Health Organization declared the pathogen a subject of international concern and the second line represents the first day of a COVID-19 case was detected in each country: February 23 in Switzerland, January 31 in Spain, February 18 in Iran, and February 24 in Pakistan. In Spain, the day that the WHO declared the pathogen of international concern was 1 day before the first case was found in Spain, and therefore the difference between the first two lines is indistinguishable in the graph. The third gray line shows the day when the WHO declared the pandemic: 11 March 2020. **(B)** Positivity rate, measured as the fraction of all tests that are positive each week. **(C)** The relationship between test intensity (measured as tests per million population) and the positivity rate each week. In Switzerland, the COVID-19 Datahub did not have testing numbers before epidemiological week 22, but the Swiss government provides those estimates for download, so we have combined both datasets [66]. In Iran, the COVID-19 Datahub did not have testing numbers before epidemiological week 15, but we knew that there would be 78,434 tests administered on week 16, and we found a publication that stated that there were about 600 tests per day done at the end of the first week after the first case, and 6000 tests performed by the end of the first month [53]. We therefore took a linear interpolant to calculate the number of tests that were done in those first 8 weeks.

Corona virus testing intensity and outcomes are shown in Figure 2. Testing began to ramp up on the week after the first case was found in all countries. The testing rate was relatively high at 4-8 thousand tests per 1 million population per week in both Switzerland and Spain, but under 2000 per 1 million population in Iran and under 1000 per 1 million population in Pakistan. The positivity rate was highest in Switzerland, Spain, and Iran during March, and fell during April and subsequent months. In Pakistan the peak positivity rate was not reached until the last week of May and first week of June. The intensity of testing had no relationship with the positivity rate in Switzerland, Iran, and Pakistan, but it had a substantial and significant relationship in Spain (*p*=<0.01, correlation coefficient r = −0.76) indicating that the high positivity rate may be attributable to the low number of tests in the first weeks after the outbreak.

## Discussion

We discuss our findings by describing the application of responses to COVID-19 and their consequent influence on the evolution of the pandemic in Switzerland, Spain, Iran, and Pakistan. We found that while *a priori* many of the systems domains delineated by the WHO were addressed in each country’s response (Figure 1; Table 3), the application of measures and their consequent influence on the evolution of the pandemic varied widely. Our findings show that the capacity of governments to sustain preventive measures was affected by different contextual factors, sometimes leading to quick resurgence in transmission.

### Switzerland: The Swiss Journey From Decentralized to Centralized Decision-Making and Back

Policy-making is heavily decentralized in Switzerland’s 26 cantons. However, during an epidemic, the Epidemics Act allows the transfer of decision-making from sub-national to national levels through escalating steps from “normal,” over “special” to “extraordinary situation” [20].

Three days after the first confirmed case on 25 February 2020, the Federal Council declared the “special situation” and banned events with more than 1000 visitors [21]. After an initial lag, decisions were made in quick succession: when Ticino, the canton with the highest disease burden, declared a “state of emergency” on 11 March 2020, several other cantons introduced stricter measures and the national government announced the closure of schools and banned events of over 100 participants. After four more cantons declared a “state of emergency”, the government escalated the national situation to “extraordinary” on 15 March 2020, enabling centralized decision-making and all shops, restaurants, entertainment facilities, and international borders were closed (Figure 1). This “extraordinary” situation allowed the government to decide on national matters without consultation of the cantons, thereby allowing for faster reactive policy-making. The centralization of the decision-making power was well received by some cantons: it provided support to contain the pandemic and required the government to take responsibility for the mandated measures and resulting economic consequences. On the other hand, some cantonal authorities criticized a lack of involvement in the strategic communication and little time to prepare before decisions were communicated to the public [22].

Even with centralized decision-making, cantons retained some decision-making capacity: they had the freedom to make their own policies if there was no national ordinance. The resulting, often complex, situation of decentralization can be exemplified by the governing council of the canton Uri, which decided on March 20 to ban people over 65 years old from leaving their houses but on the same day the Federal Council issued a new ordinance rendering the canton’s decision invalid and lifting the curfew [23, 24].

Stepwise re-opening began on 27 April 2020. On 19 June 2020, the situation was de-escalated from the “extraordinary” to “special,” returning some autonomy back to the cantons. The opening and de-escalation steps were taken more quickly than expected; cantons criticized the short timeframes between discussion and decision-making, and between communication of the decisions and implementation [22].

In 2020, the government mobilized 74 billion Swiss Francs (CHF) for combatting the pandemic in the form of loans for companies and social welfare [25]. On 25 June 2020, the Federal Council fully subsidized all tests for symptomatic persons, persons in close contact with the positive cases, and persons that were in quarantine mandated by cantonal authorities.

At the height of the first wave, wearing masks was only recommended for cases, their care takers or risk groups. The public perceived this as a strategic decision due to a shortage of masks, which was disputed by the Federal Office of Public Health (FOPH) as the scientific basis for this mandate was not yet given [26]. A report from 2021 attests that there was a severe shortage of masks by the end of February 2020 [27]. Following an increase in cases in July 2020 (Figure 1), the first national mask mandate in public transport was issued. The Swiss NationaL COVID-19 Science Task Force (SN-STF) called for stricter measures (such as a mask mandate in shops). The public discourse was dominated by a sense of uncertainty by the statements of the SN-STF, the diverging actions of the Federal Council, and the cantons’ sense of being overburdened and unsupported. At the end of July 2020, the FOPH proposed uniform national rules to avoid confusion among the public [28].

### Spain: Decentralization and Citizens’ Influence on the Response

Spain enacted surveillance and monitoring mechanisms before detecting the first confirmed case on 31 January 2020 [29]. The national government activated existing coordination mechanisms for an integrated response across ministries and regions, and created a communication strategy to raise awareness of the transmission risk and preventive measures. However, the false perception of low community transmission risk among the public resulted in low compliance with preventive measures in the initial phases of the pandemic [30]. Eventually when Spain became one of the epicenters of the health crisis in Europe, the public perception changed dramatically [31].

Despite a rapid increase in the number of cases in February, only on 3 March 2020, community transmission was declared in Madrid, Basque Country and La Rioja, and more restrictive measures (e.g., school closures and congregation restrictions) were introduced. The legal framework of the decentralized governmental system in Spain made it impossible to implement targeted lockdowns in autonomous regions and so the national government had to resort to the declaration of a nationwide lockdown on 14 March 14, 2020.

Initially, PCR tests were reserved for hospitalized patients, health professionals and workers in essential services. After 7 May 2020, testing was extended to all suspected cases and diagnosis, surveillance, and contact tracing were performed by the public health system.

Primary Health Care (PHC) was left outside of COVID-19 pandemic planning and management and the strategic focus on hospital care limited the potential of the PHC system to respond to the pandemic and led to a deficient contact tracing system in some regions [32, 33]. With the increased demand in hospitals and nursing homes, PHC providers were reallocated to provide treatment to COVID-19 patients [34]. With an overstretched PHC system and shortage of personal protective equipment (PPEs) by April 2020, Spain had the highest number of health professionals infected with COVID-19 worldwide [35].

Years of structural adjustment programs after the 2008 economic crisis in Spain left an under-resourced social and health care system [33]. Although structural and contingency funds were mobilized, and social measures to protect the most vulnerable populations were activated (e.g., guarantee home care for dependent persons), these measures could not fix the existing structural gaps. An under-resourced PHC and failing to monitor the quality of care and social services resulted in almost 20,000 deaths in nursing homes between January and June 2020 [36–39].

While public acceptance of preventive measures increased due to recognition of the epidemic’s gravity, debate over the lockdown measures rose steadily since April 2020. The lockdown in Spain was one of the strictest lockdowns in Europe resulting in negative social and economic impacts [40]. Compared to the more proactive containment strategies (massive testing and contact tracing) taken by countries such as South Korea, in early stages of the pandemic, Spain’s approach was criticized in the scientific literature as being unnecessarily restrictive in controlling the spread of the virus [41, 42]. Fear of economic slowdown and political polarization in parliament combined with social opposition to restrictive measures resulted in the loss of parliamentary support for the continued state of alarm [43, 44]. On 21 June 2020, all pandemic response competencies were fully devolved to the autonomous communities and the quick reopening resulted in deficient implementation of tracking and tracing systems, likely hindering the efficiency of the pandemic response [43–45].

### Iran: Whole of Government Approach Under Economic Pressure

Iran was among the first countries to face the heavy burden of the COVID-19 outbreak. Immediately after officially detecting the first case of the disease on 19 February 2020, the National COVID-19 Committee (NCC) was established. Among the NCC’s immediate decisions (between February 22–26, 2020) were suspending commercial flights from China, issuing health certificates for foreign travelers, closing schools and universities, banning public gatherings, congregation restrictions, and reducing working hours (Table 3).

Although all economies were significantly handicapped by the pandemic, Iran’s economy faced a double burden due to pre-existing unilateral economic sanctions; therefore, timeliness and effectiveness of mitigation strategies were overshadowed by low economic resilience [46]. Despite the growing number of cases in February 2020, authorities hesitated to impose more restrictive measures such as national lockdown, which did not come into effect until early March 2020 (Figure 1). The continuous surge in daily reported new cases to over 1000 in March 2020, combined with concerns about the high risks of spread of the virus during the Persian New Year (Nowruz) holidays on 20 March 2020, led to imposing further restrictions, such as fines for travel ban violations [47].

Eventually economic concerns and frequent changes in the lead policy-makers in the most conflicted provinces led to the premature lifting of COVID-19 restrictions. By 3 April 2020, during the peak of the first wave, businesses, which had been closed since 18 March 2020 due to Nowruz holidays, reopened gradually. Easing the preventive measures continued with the reopening of mosques, allowing religious ceremonies during Ramadan (25 April–24 May 2020), and gatherings during the consecutive holidays of Eid —celebration of the end of the month of Ramadan— which likely instigated the rise of the second wave.

With the unilaterally imposed economic sanctions, adopting a whole of government (WOG) approaches has led to the self-sufficiency of Iran in the face of shortages of the basic prerequisites for managing COVID-19. The strong political support of the Supreme Council for National Security, within the framework of the WOG approach, allowed the government to launch various national campaigns and make use of the resources of the army and many other national organizations for conducting training programs, providing health support packages, monitoring and tracking the disease.

Given the state of the fragile economy, the risk of low compliance from public to restrictive measures at national level and differences in the prevalence of COVID-19 across provinces, the NCC delegated policy-making powers for re-imposing restrictions to the provincial COVID-19 Committees [48].

Despite the efforts to minimize the economic burden, as a result of COVID-19 restrictions, about 3 million Iranians lost their jobs between March and September 2020 and the government’s financial aid to the affected businesses and households was insufficient to protect them from economic hardship [49, 50].

In response to the shortage of essential medical supplies, particularly at the outset of the pandemic, the government facilitated import, banned exports and incentivized the domestic industry to increase production capacity [51]. The capacity for real-time PCR tests increased from two centers to 190 laboratories by 22 July 2020 [52, 53], led by the Pasteur Institute of Iran, which began coordination on the first week a case was detected [53]. However, only symptomatic individuals were allowed to be tested free of charge and upon a physician’s request [47], perhaps explaining the relationship low testing intensity in the country (Figure 2).

### Pakistan: Multi-Sectoral Response for a Whole of Society Approach

A multi-sectoral response was designed through the creation of the National Coordinating Council (NCC) to manage the epidemic 3 weeks after the first case was detected—the slowest of any of the countries in our analysis [54].

Pakistan initiated preventive strategies in January 2020. One of the first containment actions taken was contact tracing of international travelers, designating quarantine houses in airports and near borders for individuals entering Pakistan to prevent community transmission; however, the state of the quarantine houses was questionable due to unsanitary conditions [55, 56].

Pakistan’s risk communication strategies included using national television programming, mobile ringtone messaging, the development of a helpline, and daily-televised briefings by the Ministry. However rumors and misinformation from social media, framing the pandemic as a conspiracy theory hindered these efforts and the country faced the challenge of low compliance by the public to the preventive measures [55].

Implementing restrictive measures such as lockdown requires taking into account the country-specific circumstances such as population structure, health needs and resources. According to Patel et.al. economically disadvantaged people are more vulnerable to COVID-19 due to poor housing conditions, no possibility to work remotely, unstable work conditions and comorbidities [57]. Thus implementing a lockdown policy without a welfare support system in a low income country could increase the unemployment rate and further drive down compliance and increase the spread of the virus [58, 59]. This also accords with the situation in Pakistan when the government announced a national lockdown for 3 weeks starting on 15 March 2020. This decision did not receive public acceptance and was criticized and violated widely due to its economic impacts on a large portion of the population. Inefficiency in implementing the national lockdown resulted in lifting the measures after 2 weeks and introducing a “smart lockdown” strategy by enforcing the lockdown, only in places with higher positivity ratio [60].

Adopting a whole-of-society approach, the government worked with the existing social safety net “Ehsaas,” to alleviate the economic burden associated with the pandemic by providing cash disbursements for daily wage earners starting in April 2020 [61].

Despite the concerns about maintaining social distancing during the prayers at mosques, the mosques were open to the public during the month of Ramadan (23 April–23 May 2020) [62]. The support of religious leaders during Ramadan was instrumental in gaining broad compliance in many areas of the country [63]. On 19 May 2020 and right before the religious festival of Eid which ends the month of Ramadan (23–24 May 2020), the Supreme Court decided to ease the measures and opened shopping centers and public transport, resulting in a sharp increase in cases in the following 2 weeks (Figure 1).

The initial shortage of health commodities and medical equipment in April and May 2020 was addressed by the disbursement of more than six billion Pakistani rupees (PKR) (USD 37M) to buy equipment, ventilators and to upgrade hospital facilities. Additionally, state banks provided low-interest loans to hospitals to improve their case management capacity [64]. While diagnostic testing was initially very scarce, Pakistan acquired increased testing capacity in late February 2020 when several testing sites across the country were established by the federal government under the supervision of Pakistan’s National Institute of Health (NIH) [65]. However, within the 4-country case study presented here, Pakistan observed a relatively low testing intensity (Figure 2), probably representative of the testing capacity of poorer countries around the world.

### Limitations

Our study is subject to a number of limitations. The accuracy of the observed incidence might not be comparable as different countries had different testing and diagnostic policies (Figure 2). There were not enough jurisdictions examined across the 17 indicators to analyze the independent and synergistic effects of each policy in a quantitative manner, and therefore we decided to rely on a structured thematic periodization of the package of interventions. Data from more countries could have also improved the geographic representation of the sample, but as this was a volunteer-based data collection effort, we relied on available and willing colleagues. Despite the collection of 17 indicators for 7 months, the decentralized responses in any one country could not be fully captured, nor could shortcomings with the centralization of decision-making. Moreover, the indicators of the economic impact of lockdowns are not perfectly comparable across countries whose informal employment sector is substantial.

### Conclusion

Health systems are complex adaptive systems embedded in a wider ecosystem of economic, social and cultural super-systems that influence each other. Disentangling the effects of this dynamic interaction to capture independent and synergistic effects of policies require both transparencies in publicly available information and a broad collection across jurisdictions of one country or several countries. The results illustrate that the functional boundaries of the health system do not stop at the edges of WHO’s six building blocks of the health systems framework.

The policy responses to COVID-19 are largely dependent on the level of decentralization of the system, their social and cultural contexts and the economic forces that define them.

Health systems with chronically under-resourced primary care and public health services, weak governance mechanisms, and substantial fragmentation across services hampered the ability of governments to respond to the health needs of citizens in a timely manner. Primary health care is the first contact point of people with the health system, however during the pandemic it was overshadowed by prioritizing secondary care. An under-resourced primary health care slowed down preventive responses and led to increased transmission.

Overall economic context and the strength of social protection systems played a crucial role in the type of interventions that the different governments put in place. Access to COVID-19 tests and functional health infrastructures allowed Switzerland to take a proactive approach to “flatten the curve” using containment measures such as testing and contact tracing thus avoiding a national lockdown. On the other side of the spectrum, in Iran and Pakistan implementing a partial lockdown was an inevitable choice, not only because of limited access to diagnostic tests but also due to the low coverage of sick or unemployment benefits.

Another major finding was that in all countries compliance to the measures was a concern. This further reinforces the importance of effective communication strategies and the need to galvanize context-driven “trust” dynamics between population and centralized and decentralized governments.
