# Supplementary material for: From Public Health Policy to Impact for COVID-19: A Multi-Country Case Study in Switzerland, Spain, Iran and Pakistan
Source: Int J Public Health. 2022 Aug 31;67:1604969. doi: 10.3389/ijph.2022.1604969 (PMC9472296; doi:10.3389/ijph.2022.1604969)
Supplement: Supplementary file 1 [file DataSheet1.DOCX]

Appendix 1. List of information sources for Figure 1

## Switzerland

1. <https://www.admin.ch/opc/de/classified-compilation/20071012/index.html>
2. <https://www.fedlex.admin.ch/eli/cc/2020/141/de>
3. https://www.efv.admin.ch/efv/en/home/aktuell/brennpunkt/covid19.html
4. Who foots the bill for the Swiss coronavirus relief package? - SWI swissinfo.ch
5. https://www.fedlex.admin.ch/eli/cc/2020/711/en
6. <https://www.admin.ch/opc/en/classified-compilation/20200744/index.html>
7. <https://www.youtube.com/playlist?list=PLEnHzNShzOwbxmvpk7ajVhE3m1mO6h6p9>
8. <https://www.bag.admin.ch/bag/en/home/das-bag/aktuell/medienmitteilungen.html?dyn_startDate=01.01.2020&dyn_endDate=01.09.2020>
9. <https://www.fedlex.admin.ch/eli/cc/2020/438/de>
10. <https://www.admin.ch/gov/en/start/documentation/media-releases.msg-id-79711.html>
11. <https://www.fedlex.admin.ch/eli/cc/2020/468/en>
12. <https://www.bag.admin.ch/bag/en/home/krankheiten/ausbrueche-epidemien-pandemien/aktuelle-ausbrueche-epidemien/novel-cov/isolation-und-quarantaene.html#-728570953>
13. <https://www.bag.admin.ch/bag/de/home/das-bag/aktuell/medienmitteilungen.msg-id-78273.html>
14. <https://www.bag.admin.ch/bag/en/home/das-bag/aktuell/medienmitteilungen.msg-id-79522.html>

## Spain

1. https://www.mscbs.gob.es/gabinete/notasPrensa.do?id=4786
2. <https://www.boe.es/diario_boe/txt.php?id=BOE-A-2020-3692>
3. [https://www.boe.es/buscar/act.php?id=BOE-A-2020-3580
   https://www.europapress.es/sociedad/educacion-00468/noticia-gobierno-abonara-septiembre-ccaa-2000-millones-educacion-80-ira-alumnos-16-anos-20200616132317.html](https://www.boe.es/buscar/act.php?id=BOE-A-2020-3580)
4. <https://www.boe.es/buscar/doc.php?id=BOE-A-2020-4174>
5. <https://www.mscbs.gob.es/profesionales/saludPublica/ccayes/alertasActual/nCov/videosPrensa0420.htm>
6. [https://www.boe.es/buscar/act.php?id=BOE-A-2020-3434; https://www.mscbs.gob.es/gabinete/notasPrensa.do?id=4856](https://www.boe.es/buscar/act.php?id=BOE-A-2020-3434)
7. https://www.dsn.gob.es/es/actualidad/sala-prensa/coronavirus-covid-19-23-febrero-2020;
8. https://boe.es/diario_boe/txt.php?id=BOE-A-2020-4929
9. https://www.boe.es/buscar/act.php?id=BOE-A-2020-3433
10. https://www.boe.es/buscar/doc.php?id=BOE-A-2020-4933
11. https://www.covid19healthsystem.org/countries/spain/livinghit.aspx?Section=1.2%20Physical%20distancing&Type=Section
12. https://www.covid19healthsystem.org/countries/spain/livinghit.aspx?Section=1.2%20Physical%20distancing&Type=Section
13. https://www.lamoncloa.gob.es/covid-19/Paginas/prevencion-e-higiene.aspx
14. https://www.covid19healthsystem.org/countries/spain/livinghit.aspx?Section=1.2%20Physical%20distancing&Type=Section
15. https://www.covid19healthsystem.org/countries/spain/livinghit.aspx?Section=1.2%20Physical%20distancing&Type=Section
16. https://www.mscbs.gob.es/gabinete/notasPrensa.do?id=4808;
17. https://www.boe.es/boe/dias/2020/05/23/pdfs/BOE-A-2020-5265.pdf
18. https://www.boe.es/boe/dias/2020/05/23/pdfs/BOE-A-2020-5265.pdf;
19. https://www.boe.es/diario_boe/txt.php?id=BOE-A-2020-5895
20. https://www.lamoncloa.gob.es/covid-19/Paginas/mapa-fases-desescalada.aspx
21. https://www.boe.es/buscar/pdf/2020/BOE-A-2020-3692-consolidado.pdf;
22. https://www.covid19healthsystem.org/countries/spain/livinghit.aspx?Section=1.2%20Physical%20distancing&Type=Section
23. https://www.boe.es/buscar/doc.php?id=BOE-A-2020-4932; https://www.boe.es/diario_boe/txt.php?id=BOE-A-2020-6508;
24. https://boe.es/diario_boe/txt.php?id=BOE-A-2020-6902;
25. https://www.boe.es/diario_boe/txt.php?id=BOE-A-2020-7140

## Iran

1. https://b2n.ir/r72303
2. https://media.coronomy.ir/uploads/org/2020/10/28/160388818171304700.pdf
3. https://cutt.ly/pyXb6sI
4. https://sptnkne.ws/BXg8
5. https://www.tabnak.ir/0042Pk,
6. https://www.isna.ir/news/98121108350/
7. https://fa.shafaqna.com/news/904388/
8. https://cutt.ly/kyYDXen
9. https://cutt.ly/gyY3oSz
10. https://cutt.ly/SyY3o8H
11. https://cutt.ly/uyY3fIm
12. https://cutt.ly/6yY3joV
13. https://cutt.ly/EyY3knn
14. https://cutt.ly/MyY3lKL
15. https://cutt.ly/kyY3zkq
16. https://cutt.ly/OyY3z6L
17. http://president.ir/fa/115338
18. www.irna.ir/news/83854642/
19. http://dolat.ir/detail/335311
20. [www.irna.ir/news/83854642/](http://www.irna.ir/news/83854642/)

Some sources are in form of pdf and can be found in [this link](https://drive.google.com/drive/folders/1ZebRZzxVQq8AAFbnMDklf7XPpoqvpMnC?usp=sharing)

## Pakistan

<https://www.covid.gov.pk/>

1. [01June2020_20200330_Guidelines_for_Shops_during_COVID19_0902.pdf](https://storage.covid.gov.pk/new_guidelines/01June2020_20200330_Guidelines_for_Shops_during_COVID19_0902.pdf)
2. [01June2020_20200326_Guidelines_for_Soial_Distancing_0601.pdf (covid.gov.pk)](https://storage.covid.gov.pk/new_guidelines/01June2020_20200326_Guidelines_for_Soial_Distancing_0601.pdf)
3. [01June2020_20200327_Guidelines_for_Zoning_of_Hospitals_0701.pdf (covid.gov.pk)](https://storage.covid.gov.pk/new_guidelines/01June2020_20200327_Guidelines_for_Zoning_of_Hospitals_0701.pdf)
4. [01June2020_20200329_Preventive_Guidelines_for_Industries_and_Workers_against_COVID19_1002.pdf](https://storage.covid.gov.pk/new_guidelines/01June2020_20200329_Preventive_Guidelines_for_Industries_and_Workers_against_COVID19_1002.pdf)
5. [09July2020_20200404_Guidelines_for_Quarantine_Facility_Establishment_0302.pdf (covid.gov.pk)](https://storage.covid.gov.pk/new_guidelines/09July2020_20200404_Guidelines_for_Quarantine_Facility_Establishment_0302.pdf)
6. [National Guidelines COVID-19 & PPEs](https://storage.covid.gov.pk/new_guidelines/01June2020_20200509-Guidance_on_selection_and_use_of_PPE.pdf)
7. [01June2020_20200529_Guidelines_for_Home_Isolation_03.pdf (covid.gov.pk)](https://storage.covid.gov.pk/new_guidelines/01June2020_20200529_Guidelines_for_Home_Isolation_03.pdf)
8. [01June2020_20200530_Guidelines_for_working_of_OPDs_for_Routine_Patients.pdf (covid.gov.pk)](https://storage.covid.gov.pk/new_guidelines/01June2020_20200530_Guidelines_for_working_of_OPDs_for_Routine_Patients.pdf)
9. [07June2020_20200607_Guidelines_for_Government_Employees_and_Organizations_3401.pdf (covid.gov.pk)](https://storage.covid.gov.pk/new_guidelines/07June2020_20200607_Guidelines_for_Government_Employees_and_Organizations_3401.pdf)
10. [Date: 26 March 2020 (covid.gov.pk)](https://storage.covid.gov.pk/new_guidelines/08June2020_2020608_Guidelines_for_mandatory_use_of_face_mask_1704.pdf)
11. [27June2020_20200627_Guidelines_to_Implement_Preventive_Health_Measures_for_Passengers_2802.pdf (covid.gov.pk)](https://storage.covid.gov.pk/new_guidelines/27June2020_20200627_Guidelines_to_Implement_Preventive_Health_Measures_for_Passengers_2802.pdf)
12. [28June2020_20200628_Guidelines_for_International_Departure_from_Pakistan_02.pdf (covid.gov.pk)](https://storage.covid.gov.pk/new_guidelines/28June2020_20200628_Guidelines_for_International_Departure_from_Pakistan_02.pdf)
13. [02July2020_20200701_National_Testing_Guidelines_for_(RT-PCR)_Diagnostic_Test_0103.pdf (covid.gov.pk)](https://storage.covid.gov.pk/new_guidelines/02July2020_20200701_National_Testing_Guidelines_for_(RT-PCR)_Diagnostic_Test_0103.pdf)

Other sources

1. <https://www.theguardian.com/world/2020/mar/19/pakistan-coronavirus-camp-no-facilities-no-humanity>
2. <http://pubdocs.worldbank.org/en/760541593464535534/World-Bank-G2Px-COVID19-Pakistan-Brief.pdf>
3. <https://theconversation.com/pakistans-religious-leaders-defied-coronavirus-mosque-restrictions-then-compromised-136941>
4. <https://apps.who.int/iris/handle/10665/353212>
5. <https://crisis24.garda.com/>
